# Antimicrobial Photodynamic Therapy: Study of Bacterial Recovery Viability and Potential Development of Resistance after Treatment

**DOI:** 10.3390/md8010091

**Published:** 2010-01-20

**Authors:** Anabela Tavares, Carla M. B. Carvalho, Maria A. Faustino, Maria G. P. M. S. Neves, João P. C. Tomé, Augusto C. Tomé, José A. S. Cavaleiro, Ângela Cunha, Newton C. M. Gomes, Eliana Alves, Adelaide Almeida

**Affiliations:** 1 CESAM and Department of Biology, University of Aveiro, Campus Universitário de Santiago, 3810-193 Aveiro, Portugal; E-Mails: anabelatavares@ua.pt (A.T.); acunha@ua.pt (A.C.); gomesncm@ua.pt (N.C.M.G.); elianaalves@ua.pt (E.A.); 2 QOPNA and Department of Chemistry, University of Aveiro, Campus Universitário de Santiago, 3810-193 Aveiro, Portugal; E-Mails: ccarvalho@ua.pt (C.M.B.C.); faustino@ua.pt (M.A.F.); jtome@ua.pt (J.P.C.T.); actome@ua.pt (A.C.T.); jcavaleiro@dq.ua.pt (J.A.S.C.)

**Keywords:** cationic porphyrin, antimicrobial photodynamic therapy, bacterial resistance, bacterial viability, bioluminescence

## Abstract

Antimicrobial photodynamic therapy (aPDT) has emerged in the clinical field as a potential alternative to antibiotics to treat microbial infections. No cases of microbial viability recovery or any resistance mechanisms against it are yet known. 5,10,15-tris(1-Methylpyridinium-4-yl)-20-(pentafluorophenyl)-porphyrin triiodide (Tri-Py^+^-Me-PF) was used as photosensitizer. *Vibrio fischeri* and recombinant *Escherichia coli* were the studied bacteria. To determine the bacterial recovery after treatment, Tri-Py^+^-Me-PF (5.0 μM) was added to bacterial suspensions and the samples were irradiated with white light (40 W m^−2^) for 270 minutes. Then, the samples were protected from light, aliquots collected at different intervals and the bioluminescence measured. To assess the development of resistance after treatment, bacterial suspensions were exposed to white light (25 minutes), in presence of 5.0 μM of Tri-Py^+^-Me-PF (99.99% of inactivation) and plated. After the first irradiation period, surviving colonies were collected from the plate and resuspended in PBS. Then, an identical protocol was used and repeated ten times for each bacterium. The results suggest that aPDT using Tri-Py^+^-Me-PF represents a promising approach to efficiently destroy bacteria since after a single treatment these microorganisms do not recover their viability and after ten generations of partially photosensitized cells neither of the bacteria develop resistance to the photodynamic process.

## 1. Introduction

The use of antibiotics to destroy selectively microorganisms (MO) represents one of the most revolutionary progresses made in scientific medicine, resulting in the treatment and sometimes complete eradication of earlier incurable diseases [1,2]. It might have been supposed that at the beginning of the twenty first century, microbiologically-based diseases would have been reduced to a level that no longer had a serious impact on human health. However, bacteria have developed resistance mechanisms against antimicrobial drugs which were previously highly effective. Besides, bacteria replicate very rapidly and a mutation that helps a MO to survive in the presence of an antibiotic will quickly become predominant in the microbial population [1,3]. Due to resistance to all β-lactam antibiotics, the glycopeptide antibiotic vancomycin has remained as last line of defense against Gram-positive bacteria. However, methicillin-resistant *Staphylococcus aureus* and vancomycin-resistant *enterococci* are species that are causing much concern at present [4] and there is an urgent need for the development of novel, convenient, non-resistant and inexpensive measures for fighting microbial diseases [1,5,6].

Antimicrobial photodynamic therapy (aPDT) represents a potential alternative methodology to inactivate microbial cells [7–9] and has already shown to be effective *in vitro* against bacteria, fungi, viruses and protozoa [5,10–17]. The aPDT approach is based on the photodynamic therapy concept that comprises the action of three components: a photosensitizing agent (PS), a light source of an appropriate wavelength (artificial light or sunlight) and oxygen [1,5,18–20]. Two oxidative mechanisms of photoinactivation (PI) are considered to be implicated in the inactivation of the target cells. The type I pathway involves electron/hydrogen atoms-transfer reactions from the PS triplet state with the participation of a substrate to produce radical ions while the type II pathway involves energy transfer from that triplet state to molecular oxygen to produce singlet oxygen (^1^O_2_) [3,5,21–23]. Both processes lead to highly toxic reactive oxygen species (ROS) such as ^1^O_2_ and free radicals, able to irreversibly alter vital components of cells resulting in oxidative lethal damage [24,25]. The main advantages of aPDT are the non-target specificity, the few side effects, the prevention of the regrowth of the MO after treatment and the lack of development of resistance mechanisms due to the mode of action and type of biochemical targets (multi-target process) [9,20].

The photodynamic activity produces damages mainly in the cytoplasmatic membrane and in DNA [3]. The damages to the cytoplasmatic membrane can involve leakage of cellular contents or inactivation of membrane transport systems and enzymes [26,27]. Some damages produced in the DNA chain can be repaired by the action of DNA repairing systems [28]. However, it was shown that although DNA damage occurs it cannot be the main cause of bacterial cell photodynamic inactivation [3,29], since *Deinococcus radiodurans*, which is known to have a very efficient DNA repair mechanism, is easily killed by aPDT [30].

Although various studies have investigated the possible recovery of bacterial infections in animal models (*in vivo*) [31–33], there are no published results from studies that tested the possible viability recovery, *in vitro*, after aPDT treatment. Moreover, despite the fact various authors have stated that resistance to aPDT is unlikely to occur due to the non-specific killing mechanism (ROS cause damage on diverse bacterial structures) [34–41], only a few studies were conducted in order to determine if bacterial resistance occurs after several consecutive aPDT treatments. Cell wall structures and membranes are the main targets of photodynamic therapy drugs, and for this reason the drugs do not necessarily need to enter the cell. Specific and proper adhesion to these structures is usually considered sufficient for light-activated destruction of the target cell. Thus target cells have no chance to develop resistance by stopping uptake, increasing metabolic detoxification or increasing export of the drug [9].

The research concerning aPDT is more focused in the identification of new PS that kill rapidly and efficiently the MO and in the disclosure of their inactivation mechanisms. However, and regarding the emergence of bacterial resistance to antibiotics, it is important to control the PI process in terms of resistance development. Lauro *et al*. [42] investigated the selection of resistant bacterial strains in *Peptostreptococcus micros* and *Actinobacillus actinomycetemcomitans* after repeated photosensitization of surviving cells with the porphycene-polylysine conjugates 2,7,12,17-tetrakis(2-methoxyethyl)-9-glutaramidoporphycene and 2,7,12,17-tetrakis(2-methoxyethyl)-9-*p*-carboxybenzyl-oxyporphycene. The results obtained by this group showed that the photosensitization of *P. micros* and *A. actinomycetemcomitans* by both PS induced no appreciable development of resistance in partially inactivated bacterial cells. The efficiency of photokilling underwent no change in ten subsequent irradiation sessions, even though cells which were damaged in a previous treatment were cultivated and re-exposed to porphycene and light [42]. Pedigo *et al*. [43] studied the possible development of bacterial resistance to aPDT after several treatments in antibiotic sensitive (MSSA) and resistant strains (MRSA) of *S. aureus* and antibiotic sensitive *Escherichia coli*. Bacteria were exposed to repetitive aPDT treatments using methylene blue as PS. The parameters were adjusted such that photoinactivation were lowest than 100% so that surviving colonies could be employed for subsequent exposures. No significant difference in *E. coli* cell death was observed through eleven repeated aPDT treatments. Similar results were observed using MSSA and MRSA, for which the killing rate did not significantly differ from over twenty five repeated exposures [43]. Jori *et al.* [44] determined that up to five consecutive generations of extensively photoinactivated MRSA (ca. 90%) show essentially identical degrees of sensitivity to phthalocyanine photosensitization. Although the known studies indicate that bacterial resistance to aPDT is unlikely, it is an important parameter to be evaluated when a new PS is considered for aPDT.

The bacterial bioluminescence method is considered to be a rapid, sensitive and cost-effective choice [12,45–47] to monitor the possible development of resistance after consecutive aPDT treatments, once that the photodynamic activity can be measured directly, continuously and in a nondestructively high-throughput screening or continuous-culture way [48]. A strong correlation between bioluminescence and viable counts was demonstrated in experimental systems [12,49,50], where the light output reflects the actual cells metabolic rate.

**Figure 1:**
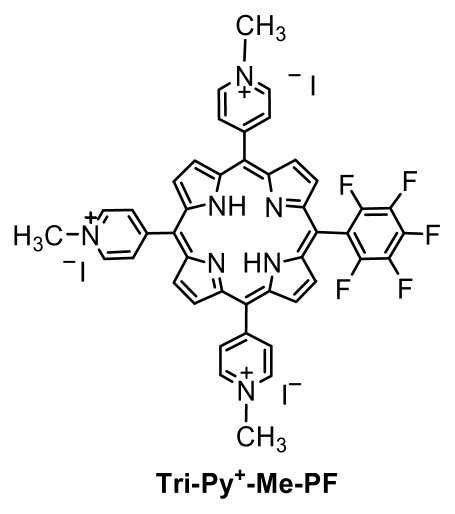
The structure of 5,10,15-tris(1-methylpyridinium-4-yl)-20-(pentafluorophenyl)-porphyrin triiodide.

The aim of this study is to determine if in the presence of 5,10,15-tris(1-methylpyridinium-4-yl)-20-(pentafluorophenyl)porphyrin triiodide (Tri-Py^+^-Me-PF; Figure 1) the bacterial cells can recover their activity after photodynamic treatment and develop resistance to aPDT after repeated photodynamic treatments. The Tri-Py^+^-Me-PF is a tricationic porphyrin recently described by our group. We have shown that it is a promising PS for the inactivation of several types of MO [10–12,16,17,51].

To achieve the goals indicated above, the bacterial bioluminescent method was selected to monitor the bacterial activity of two bioluminescent Gram-negative bacteria: the non-transformed *Vibrio fischeri* and the recombinant *Escherichia coli*.

## 2. Results and Discussion

### 2.1. Results

The bacterial strains used in this work were a recombinant bioluminescent strain of *E. coli* described in a previous work [12] and the bioluminescent marine bacterium *Vibrio fischeri* ATCC 49387. Knowing that the light emission in these bacteria is directly proportional to their metabolic activity [12], we used their bioluminescence ability to evaluate their recovery after photodynamic treatment. The possible development of bacterial resistance after several aPDT treatments with Tri-Py^+^-Me-PF as PS was also evaluated using the bioluminescence method.

#### 2.1.1. Bioluminescence versus CFU of an overnight culture

**Figure 2:**
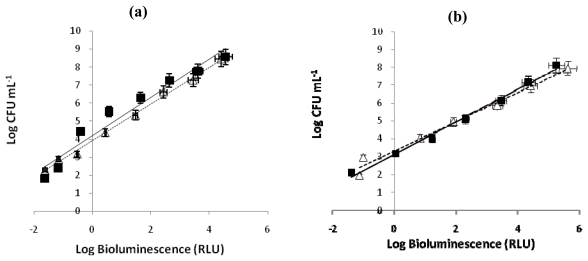
(a) Relationship between the bioluminescence signal and viable counts of overnight cultures of bioluminescent marine bacterium *Vibrio fischeri* (≈10^9^ CFU mL^−1^) serially diluted in PBS with 3% of NaCl. (b) Relationship between the bioluminescence signal and viable counts of overnight cultures of recombinant bioluminescent *Escherichia coli* (≈10^8^ CFU mL^−1^) serially diluted in PBS. Viable counts are expressed in CFU mL^−1^ and bioluminescence in relative light units (RLU). The values are expressed as the means of two independent experiments; error bars indicate the standard deviation. (--▵-- bacterial suspension in the absence of PS, —▪— bacterial suspension with 5.0 μM of Tri-Py^+^-Me-PF incubated 4h in the dark).

To evaluate the correlation between the colony-forming units (CFU) and the bioluminescence signal of *V. fischeri* and *E. coli*, the assays were carried out in dark conditions, with and without Tri-Py^+^-Me-PF porphyrin. A linear correlation between viable counts and the bioluminescence signal of overnight cultures of both bioluminescent strains was observed (Figure 2). These correlations are similar in the presence and in the absence of Tri-Py^+^-Me-PF and the bioluminescence results reflect the viable bacterial abundance.

#### 2.1.2. aPDT recovery study

**Figure 3:**
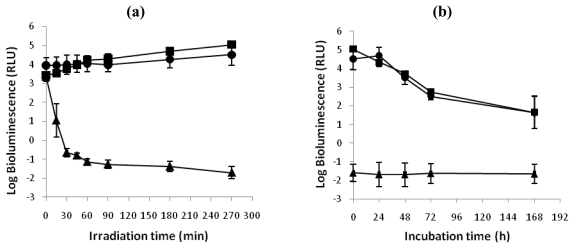
(a) Bioluminescence monitoring of *V. fischeri* treated with Tri-Py^+^-Me-PF at 5.0 μM after 15, 30, 45, 60, 90, 180 and 270 minutes of irradiation with white light (40 W m^−2^). (b) Bioluminescence signal of *V. fischeri* 24, 48, 72 and 168 hours after the photodynamic treatment. The values are expressed as the means of two independent experiments; error bars indicate the standard deviation. (-▪-dark control, -^- light control, -▴- Tri-Py^+^-Me-PF + light).

**Figure 4:**
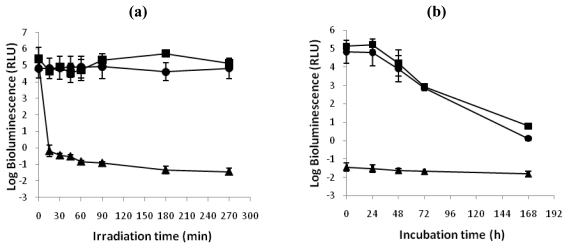
(a). Bioluminescence monitoring of *E. coli* treated with Tri-Py^+^-Me-PF at 5.0 μM after 15, 30, 45, 60, 90, 180 and 270 minutes of irradiation with white light (40 W m^−2^). (b) Bioluminescence signal of *E. coli* 24, 48, 72 and 168 hours after the photodynamic treatment. The values are expressed as the means of two independent experiments; error bars indicate the standard deviation. (-▪- dark control, -^- light control, -▴- Tri-Py^+^-Me-PF + light).

The ability of *V. fischeri* and *E. coli* cells to recover metabolic activity after a photodynamic treatment with 5.0 μM of Tri-Py^+^-Me-PF is represented in Figure 3 and Figure 4, respectively. After 270 minutes of irradiation, the limits of detection for both bacteria were reached and a reduction of 5.1 log units on the bioluminescence signal of *V. fischeri* and 6.4 log units for bioluminescent *E. coli* was observed. Moreover, light and dark controls showed that the viability of these bacteria was neither affected by irradiation itself nor by the Tri-Py^+^-Me-PF in dark conditions (Figures 3A and 4A). This confirms that the reductions obtained on cell viability after irradiation of the treated samples are due to the photosensitizing effect of the porphyrin.

After one week of incubation in culture medium in dark conditions and in growth medium (Figures 3B and 4B), it was observed that the bioluminescence signal of photodynamic treated samples of *V. fischeri* and *E. coli* was unchanged during all period of incubation (log bioluminescence ≈−1.5 RLU for both *V. fischeri* and *E. coli* cells). No colonies were observed in the non-diluted (10^0^) treated aliquot after plating in TSA medium with or without addition of NaCl, which is consistent with the bioluminescence results. It was also observed a decrease on the bioluminescence signal of light and dark controls of these bacteria.

#### 2.1.3. aPDT resistance study

**Figure 5:**
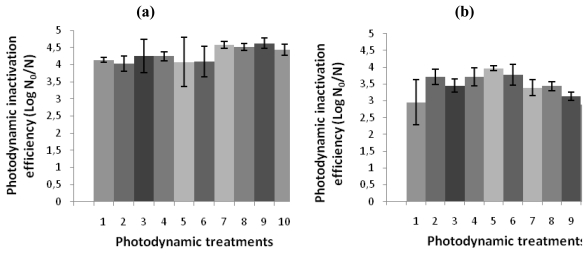
Photodynamic inactivation efficiency of ten consecutive generations of (a) *V. fischeri* and (b) *E. coli* by 5.0 μM of Tri-Py^+^-Me-PF after 25 min of irradiation with white light (40 W m^−2^), using the bacterial bioluminescence method. N_0_ and N represent, respectively, bioluminescence signal before and after the irradiation [42]. The values are expressed as the means of three independent experiments; error bars indicate the standard deviation.

The effect of repeated treatments of bacterial suspensions of *V. fischeri* or *E. coli* with 5.0 μM of Tri-Py^+^-Me-PF to white light (40 W m^−2^) for 25 minutes are represented in Figure 5. The PI efficiency by Tri-Py^+^-Me-PF against both strains was not affected during the ten generations of photosensitized cells. After 25 min of irradiation at 40 W m^−2^ the Tri-Py^+^-Me-PF was able to destroy 99.99% of *V. fischeri* and *E. coli* cells.

### 2.2. Discussion

Microorganisms have adopted a variety of mechanisms to increase their resistance to antimicrobial drugs. These mechanisms comprise a thickening of their outer wall, encoding of new proteins which prevent the penetration of drugs, and onset of mutants deficient in porin channels allowing the influx of externally added chemicals [52–54]. The emergence of antibiotic resistance by pathogenic MO has led to the search of efficient alternative methods for which mechanisms of resistance must not occur [3,55]. The aPDT represents a potential approach to inactivate pathogenic MO [7,8] and has already shown to be efficient against bacteria, yeasts, viruses, and protozoa [5,10–16,18]. The main advantages of aPDT are the MO non-target specificity, the few side effects, the prevention of the regrowth of the MO after treatment and the potential lack of development of resistance mechanisms due to a multi-target process [9,20].

The results of this study shown that both bacteria when treated with the tricationic porphyrin Tri-Py^+^-Me-PF and irradiated with white light (40 W m^−2^) for 270 minutes are unable to recover metabolic activity. When samples were maintained for one week in dark conditions and under medium growth, after the photodynamic treatments, the bioluminescence signal remains the same for both *V. fischeri* and *E. coli* cells during all period of incubation (≈−1.5 log RLU). The bioluminescence signal reduction observed in dark and light controls can be due to the lack of nutrients and/or the accumulation of toxic products from bacteria metabolism. It was also clear that the results obtained in this study corroborate the literature [42–44] relative to the absence of resistance mechanisms after the photodynamic process. In fact, no significant reduction in the efficiency of photosensitization of *V. fischeri* and *E. coli* was observed after ten consecutive photosensitization sessions of 25 minutes with 5.0 μM of Tri-Py^+^-Me-PF. If bacterial resistance would occur, important reductions on the bacterial photoinactivation efficiency would be detected between experiments (and the irradiation time required to reach 99.99% of photosensitized cells should increase). Various authors affirmed that cultures on stationary phase show a certain degree of resistance to the PS [34,56]. However, others refer that bacteria susceptibility to PS is independent from the growth phase [57,58]. In this study, the cultures were used at the same growth phase for all assays (stationary growth phase). As the bacterial colonies have been aseptically removed from the plate and resuspended in PBS, the cellular density obtained after the colony resuspension could be different. To avoid differences in the PI efficiency due to different bacterial densities, this parameter was controlled in all the experiments by measuring the optical density of the bacteria suspension before each assay.

## 3. Experimental Section

### 3.1. Photosensitizer

The tricationic porphyrin used in this work [5,10,15-tris(1-methylpyridinium-4-yl)-20-(penta-fluorophenyl)porphyrin triiodide, Tri-Py^+^-Me-PF] was prepared in two steps according to the literature [59]. A 500 μM stock solution of Tri-Py^+^-Me-PF porphyrin in DMSO was prepared and maintained at 4 °C.

### 3.2. Bacterial strains and growth conditions

The bacterial strains used in this work were a recombinant bioluminescent strain of *E. coli* prepared in a previous work [12] and the bioluminescent marine bacterium *Vibrio fischeri* ATCC 49387. Both *E. coli* and *V. fischeri* were stored at −80 °C in 10% glycerol. Before each assay, an aliquot of *V. fischeri* was aseptically plated on tryptic soy agar (TSA, Merck) complemented with 3% of NaCl (because of the osmotic pressure required to natural light emission to occur) and grown for one day at 25 °C. Next, one colony was aseptically inoculated on 30 mL Luria-Bertani broth with saline medium (LBS; 10 g of tryptone, 5 g of yeast extract and 30 g NaCl per liter; pH 7.5) [60] and grown for one day at 25 °C under stirring (100 rpm). An aliquot of this culture (240 μL) was subcultured in 30 mL of LBS and grown overnight at 25 °C under stirring (100 rpm). The same procedure was carried out with an *E. coli* strain containing two plasmids that confer resistance to two antibiotics: ampicilin (Amp) and chloramphenicol (Cm). For that reason, *E. coli* was aseptically plated on TSA with 100 mg mL^−1^ of Amp and 25 mg mL^−1^ of Cm and grown for one day at 25 °C. Next, one colony was aseptically inoculated on tryptic soy broth (TSB, Merck) with both antibiotics and grown for one day at 25 °C under stirring (100 rpm). Then, an aliquot of this culture was subcultured in 30 mL of TSB with Amp and Cm and grown overnight at 25 °C under stirring (100 rpm).

### 3.3. Irradiation conditions

The studies were carried out by exposing the samples to white light (PAR radiation, 13 lamps OSRAM 21 of 18 W each one, 380–700 nm) with a fluence rate of 40 W m^−2^ (measured with a radiometer LI-COR Model LI-250). As *V. fischeri* emits light at temperatures below 30 °C [61], the samples were placed on a tray with water in order to maintain the samples at a constant temperature (25–28 °C).

### 3.4. Bioluminescence versus CFU

To evaluate the correlation between the CFU and the bioluminescence signal of *V. fischeri*, two assays were carried out in dark conditions, with and without Tri-Py^+^-Me-PF porphyrin. Two suspensions were prepared from an overnight culture of *V. fischeri*, diluting the culture (1:10) in fresh phosphate buffered saline with 3% of NaCl (PBS with 3% of NaCl: 30 g NaCl, 0.2 g KCl, 1.44 g Na_2_HPO_4_ and 0.24 g KH_2_PO_4_ per liter; pH 7.4) to a final concentration of 10^7^ CFU mL^−1^. In one of these bacterial suspensions, an appropriate volume of porphyrin was added to achieve a final concentration of 5.0 μM, followed by a dark incubation for 4 h at 25–28 °C under stirring. Next, both suspensions were serially diluted (10^−1^–10^−7^) in PBS with 3% of NaCl. The non-diluted (10^0^) and the diluted aliquots were plated in TSA with 3% of NaCl (100 μL) and, simultaneously, were read on a luminometer (500 μL) (TD-20/20 Luminometer, Turner Designs, Inc., USA) to determine the bioluminescence signal. The correlation between the CFU and the bioluminescence signal of *E. coli* was evaluated following the procedure described above for *V. fischeri*. However, the cultures and suspensions were diluted in PBS (PBS; 8 g NaCl, 0.2 g KCl, 1.44 g Na_2_HPO_4_ and 0.24 g KH_2_PO_4_ per liter; pH 7.4) and pour plated in TSA without NaCl. Both experiments were done in duplicate and the results were averaged.

### 3.5. aPDT recovery study

A photodynamic inactivation assay was conducted in order to determine if bacterial cells can recover their metabolism after an effective treatment. For this purpose, cultures of *V. fischeri* and *E. coli* were grown overnight and diluted tenfold in PBS (supplemented with 3% NaCl in the case of *V. fischeri*) to a final concentration of 10^7^ CFU mL^−1^. These bacterial suspensions were equally distributed in 600 mL sterilized and acid-washed beakers. Afterwards, the appropriate volume of Tri-Py^+^-Me-PF solution was added to achieve a final concentration of 5.0 μM (total volume in the beakers was 15 mL). The samples were protected from light with aluminum foil and incubated for 10 min at 25–28 °C, under stirring (100 rpm), to promote the porphyrin binding to bacterial cells. Then, the mixtures were exposed to white light with a fluence rate of 40 W m^−2^ for 270 minutes (corresponding to a light fluence of 64.8 J cm^−2^) under 100 rpm (25–28 °C). Aliquots of treated and control samples were collected at time 0 and after 15, 30, 45, 60, 90, 180 and 270 min of light exposure and the bioluminescence signal was measured in the luminometer. After 270 min of irradiation, when all bacteria were inactivated to the detection limit of the method, the samples were protected from light with aluminum foil and maintained under stirring at 25–28 °C. Aliquots of samples irradiated for 270 min and control samples were collected after 24, 48, 72 and 168 hours post-irradiation and the bioluminescence signal was measured in the luminometer. Light and dark controls were included in all experiments. In the light control no Tri-Py^+^-Me-PF was added, but the beaker was exposed to the same irradiation protocol. In the dark control, the Tri-Py^+^-Me-PF was added to achieve a final concentration of 5.0 μM and it was covered with aluminum foil. Both experiments were done in duplicate and the results were averaged.

### 3.6. aPDT resistance study

In order to assess the possible development of resistance in bacterial cells after photosensitized inactivation, cultures of *V. fischeri* and *E. coli* grown overnight were centrifugated at 10,000 *g* for 15 minutes to remove dead cells and residues of culture medium, and the cells thus obtained were resuspended in PBS (supplemented with 3% of NaCl in the case of *V. fischeri*). Bacterial suspensions (10^7^ CFU mL^−1^) were equally distributed in 600 mL sterilized and acid-washed beakers and the suitable volume of Tri-Py^+^-Me-PF solution was added to achieve a final concentration of 5.0 μM (total volume was 10 mL per beaker). The samples were protected from the light with aluminum foil and incubated for 10 min at 25 °C, under stirring (100 rpm), to promote the porphyrin binding to bacterial cells. Afterwards, the mixtures were exposed to white light with a fluence rate of 40 W m^−2^ (under stirring at 25–28 °C) for 25 minutes. In that way, ca. 1 log unit of surviving bacteria is achieved. At the end of each treatment, an aliquot of both samples was plated on TSA and the plates were incubated for 48 hours at 25 °C in the case of *V. fischeri* and overnight at 25 °C for *E. coli*. Three surviving colonies from a plate of each bacterium were collected and each one was centrifugated at 10,000 *g* (15 minutes), resuspended in PBS and inoculated on the suitable liquid medium. Subsequently, the bacterial suspensions were incubated in the dark with the PS and exposed to visible light using an identical irradiation protocol that was repeated for ten times for each bacterium. Before each assay, the optical densities (O.D.) of cultures of *V. fischeri* and *E. coli* were controlled and monitored to O.D. of 0.5 and 1.3 (660 nm), approximately. The PI efficiency was expressed as log N_0_/N, where N_0_ and N represent the bioluminescence signal before and after the irradiation, respectively [42].

## 4. Conclusions

This study shows that the promising photosensitizer Tri-Py^+^-Me-PF is able to destroy efficiently Gram-negative bacteria, after the photodynamic treatment, without the recovery of bacterial viability. It was also confirmed that the bacteria photosensitized by this photosensitizer do not develop resistance mechanisms against the photodynamic process.
